# Improving intraoperative temperature management in elective repeat cesarean deliveries: a retrospective observational cohort study

**DOI:** 10.1186/s13037-020-00241-x

**Published:** 2020-04-19

**Authors:** Amie L. Hoefnagel, Kristen L. Vanderhoef, Anwar Anjum, Venkata Damalanka, Saurin J. Shah, Carol A. Diachun, Paul D. Mongan

**Affiliations:** 1 Department of Anesthesiology, University of Florida College of Medicine – Jacksonville, 655 West 8th Street; Box C-72, Jacksonville, FL 32209 USA; 2 Department of Anesthesiology & Pain Medicine, University of Washington, Seattle, WA USA

**Keywords:** Inadvertent hypothermia, Cesarean section, Quality improvement, Forced air warming, Anesthesia, Obstetrical, Body temperature, MH - cesarean section/*methods, Intraoperative complications/*prevention & control, Perioperative care

## Abstract

**Background:**

Inadvertent perioperative hypothermia (< 36 °C) occurs frequently during elective cesarean delivery and most institutions do employ perioperative active warming. The purpose of this retrospective observational cohort study was to determine if the addition of preoperative forced air warming in conjunction with intraoperative underbody forced air warming improved core temperature and reducing inadvertent perioperative hypothermia during elective repeat elective cesarean delivery with neuraxial anesthesia.

**Methods:**

We evaluated the addition of perioperative active warming to standard passive warming methods (preheated intravenous/irrigation fluids and cotton blankets) in 120 parturients scheduled for repeat elective cesarean delivery (passive warming, n = 60 vs. active + passive warming, n = 60) in a retrospective observational cohort study. The primary outcomes of interest were core temperature at the end of the procedure and a decrease in inadvertent perioperative hypothermia (< 36 °C). Secondary outcomes were surgical site infections and adverse markers of neonatal outcome.

**Results:**

The mean temperature at the end of surgery after instituting the active warming protocol was 36.0 ± 0.5 **°**C (mean ± SD, 95% CI 35.9–36.1) vs. 35.4 ± 0.5 **°**C (mean ± SD, 95% CI 35.3–35.5) compared to passive warming techniques (*p* <  0.001) and the incidence of inadvertent perioperative hypothermia at the end of the procedure was less in the active warming group - 68% versus 92% in the control group (*p* <  0.001). There was no difference in surgical site infections or neonatal outcomes.

**Conclusions:**

Perioperative active warming in combination with passive warming techniques was associated with a higher maternal temperature and lower incidence of inadvertent perioperative hypothermia with no detectable differences in surgical site infections or indicators of adverse neonatal outcomes.

## Introduction

Inadvertent perioperative hypothermia in the general surgical population (core temperature <  36 **°**C) is associated with complications ranging from bleeding and cardiac dysfunction to increased infections [1–3]. Inadvertent perioperative hypothermia occurs in 60–90% during cesarean delivery as a result of peripheral vasodilation, diminished regulatory vasoconstriction, and reduced shivering responses that promote heat redistribution during neuraxial anesthesia [4–8]. Furthermore, this may also result in decreases in neonatal temperature, umbilical blood pH, Apgar scores and associated adverse outcomes [6, 9–11]. Reducing the incidence of inadvertent perioperative hypothermia is a quality target by the Centers for Medicare & Medicaid Services and the British National Institute of Health and Care Excellence [12]. While national obstetrical organizations have not addressed this concern, the Enhanced Recovery After Surgery Society recommends active warming in addition to passive warming strategies during cesarean delivery [6].

Numerous publications have documented favorable outcomes with forced-air warming, underbody warming blankets, and warmed intravenous/irrigation fluids [5, 11, 13–17]. Inadvertent perioperative hypothermia is still common with intraoperative active warming and may be further ameliorated with 15–30 min of preoperative active warming [9, 18, 19].

In this study, we expanded our standard surgical active warming practice of preoperative (Bair Paws™ Standard Warming Gown) and intraoperative active warming (Bair full access underbody blanket) for parturients undergoing repeat cesarean delivery with neuraxial anesthesia. The primary outcome measures were maternal temperature and incidence of inadvertent perioperative hypothermia (< 36 °C).

## Methods

This retrospective observational cohort study was approved by the University of Florida-Jacksonville Institutional Review Board and waiver for individual patient consent was granted [20]. Our primary objectives were to determine if standardized implementation of pre- and intraoperative active warming (combined with warmed IV/irrigation fluids) versus standard passive warming positively impacted core temperature and the incidence of inadvertent perioperative hypothermia in parturients scheduled for elective repeat cesarean delivery. Secondary outcomes included wound complications, Apgar scores, and fetal cord blood pH and base excess. Data collection for baseline state of inadvertent perioperative hypothermia occurred between January and May 2017 and the active warming phase was instituted between June and October 2017. No industry funding supported this project and this manuscript adheres to the Squire 2.0 guidelines for reporting [21].

We evaluated 120 parturients undergoing elective repeat cesarean delivery at the University of Florida-Jacksonville Labor and Delivery Suite - an urban safety-net hospital - between January and September of 2017. Exclusion criteria were patients: < 18 years of age; active labor; a diagnosis of abnormal placentation; preeclampsia or eclampsia; significant cardiopulmonary comorbidities; temperature > 37 °C, primary cesarean delivery or a diagnosis of/or clinical evidence suggesting a disorder of coagulation or infection. A sample size power analysis was performed using mean temperature differences (0.5 °C) and SD (0.4–0.5) from studies evaluating passive warming and active warming during cesarean delivery [5, 14, 18, 22]. Using an alpha = 0.05 and power = 0.80, and an unpaired two-tailed Student’s t-test the projected sample size (GPower 3.1) was 50 (large effect size) to 60 (moderate effect size) for each group [23, 24].

### Temperature management

From January to the end of May 2017, we did not alter our routine standard passive warming practices in the preoperative and operative environment. Fresh warmed cotton blankets (40 °C) were offered in the preoperative unit. The labor and delivery operating room ambient temperature was set at 18–20 °C. Irrigation and intravenous fluids were prewarmed in a temperature-controlled cabinet (40 °C). Fresh warmed cotton blankets (40 °C) were used over the shoulder and arms in the operating room before initiation of the neuraxial block. Additional warmed blankets were used on the upper chest and arms after positioning for surgery. These were replaced at the request of the patient during the procedure and rearranged to facilitate skin to skin bonding after delivery.

During the active warming phase (June–October 2017), we replaced all warmed cotton blankets with the 3 M™ Bair Paws™ System model 875 (3 M Center, Building 275-4E-01 St. Paul, MN 55144–1000) and a Bair Paws™ patient warming gown (81003) preoperatively (40 °C) for 30–60 min before transfer to the operating room. Patients adjusted the unit to their preferred level of comfort. and were able to express thermal discomfort in the operating room allowing for adjustment of the temperature of the underbody warming blanket by the anesthesia team.

In the operating room the Bair Paws™ patient warming gown was left in place, but disconnected and rearranged to facilitate the neuraxial block and skin to skin infant bonding. After induction of neuraxial anesthesia, surgical preparation, and draping, the 3 M™ Bair Hugger™ System model 775 and a full access underbody blanket (54500) set at 43 °C was used for active warming. No warmed cotton blankets were used in the OR during this period and patients were able to express thermal discomfort in the operating room allowing for adjustment of the temperature of the underbody warming blanket by the anesthesia team. In addition, the anesthesia team could reduce the delivered temperature based on their clinical judgment or if the foley temperature exceeded 37 °C (consistent with our standard practice for general anesthetic cases using a forced air warming device).

### Anesthetic and operative management

In the operating room, after positioning the patient seated and obtaining baseline blood pressure, heart rate, and oxygen saturation, the lower back was disinfected with povidone iodine solution and a sterile fenestrated drape placed. Either a single shot spinal (PENCAN® B. Braun Medical Inc. 824 12th Avenue, Bethlehem, Pennsylvania 18,018) or combined spinal epidural anesthetic (ESPOCAN® B. Braun Medical Inc.) was established using 12 mg hyperbaric bupivacaine, 20 mcg preservative free fentanyl, and 150 mcg preservative free morphine.

Immediately after the initiation of neuraxial anesthesia, the patient was positioned supine with left uterine displacement and a Foley catheter inserted for measurement of core temperature. Fluid loading was not performed before or during the neuraxial anesthetic. Blood pressure target greater than 110 mmHg (or within 20% of baseline for patients with gestational hypertension) was primarily accomplished by a low dose phenylephrine infusion (50 mcg/min) initiated at the start of the anesthetic [25]. In addition, weight based administration of intravenous cephazolin (2 g < 120 kg or 3 g > 120 kg) was infused 60 min before skin incision.

Physiological measurements were recorded using a Nihon Koden BSM-6000 Monitor (Nihon Koden America 15,353 Barranca Parkway, Irvine, CA 92618) with automated electronic storage to the Epic® electronic health record (Epic, 1979 Milky Way, Verona, Wisconsin 53,593). Fluid therapy and medication administration were entered directly into the Epic® anesthesia record. In addition to the time entering the room and placement of the neuraxial block, other times recorded were skin incision, uterine incision, delivery, placenta delivery, and time of exit from the operating room.

After birth, umbilical arterial blood was sampled for pH and base excess. In addition, a pediatrician determined Apgar scores at 1 and 5 min. Neonatal birth weight was recorded.

### Temperature measurements

In the preoperative and postoperative recovery area the patient’s sublingual temperature was measured with a Welch Allyn Sure Temp Plus 690 oral thermometer (Welch Allyn Inc., Corporate Headquarters, 4341 State Street Road, Skaneateles Falls, NY 13153, calibration accuracy 0.1 °C at 36.0 °C). The operating room temperature was continuously measured via the T2 input of the Nihon Koden BSM-6000 using an ambient temperature probe. Core temperature was measured as soon as clinically feasible using a 14 Fr Bardex I.C. 400 Series temperature sensing Foley catheter (Bard Medical Division, 8195 Industrial Blvd. Covington, GA 30014, accuracy +/− 0.2 °C at 37.0 °C). A Foley temperature probe was chosen for evaluation of temperature due to its superior accuracy to lingual and temporal artery scanning and comparative accuracy with tympanic membrane temperatures without the risk of discomfort or trauma from a tympanic membrane temperature probe [26–28].

### Statistical analysis

Data was analyzed with IBM® SPSS® and descriptive statistics expressed as mean ± standard deviation, median (range), confidence interval, numbers, or percent as appropriate. Differences between groups for continuous, normally distributed variables were analyzed with an unpaired two-tailed Student’s t-test (Kolmogorov-Smirnoff two sample test if the data was not normally distributed). Categorical variables and proportions were analyzed with chi-square or Fisher’s exact tests. A *p* value < 0.05 was considered significant.

## Results

**Fig. 1:**
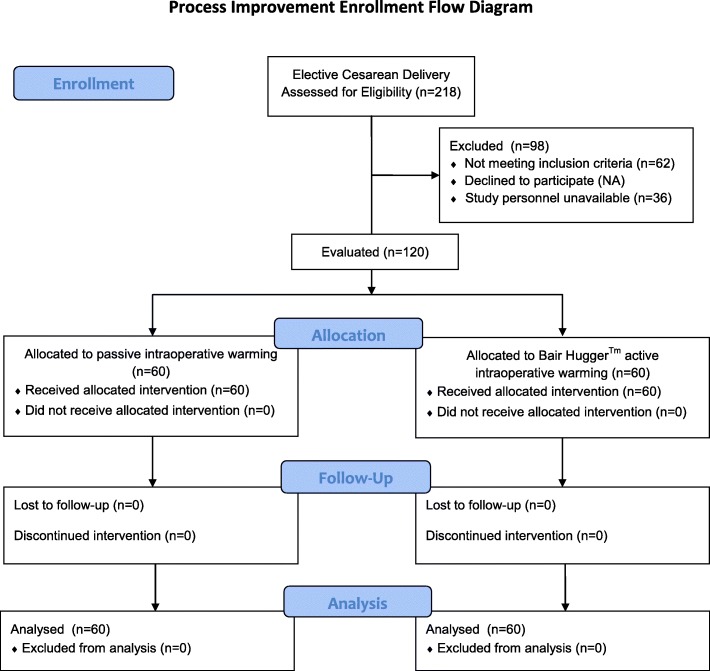
Process Improvement Enrollment Flow Diagram

Data was collected on 120 patients (Fig. 1). Table 1 lists the demographic, patient descriptors, surgical times, and intraoperative fluid volumes. During the study period there were 218 elective cesarean delivery performed. Ninety-eight patients were excluded from the evaluation due to inclusion criteria (62) or lack of personnel/equipment for implementation of active warming (36). There were no differences in maternal, gestational, or operative characteristics. All parturients were scheduled for repeat cesarean delivery with some parturients having bilateral tubal ligation and a small number of patients for repeat cesarean delivery involving multiple gestations (Table 1). The proportion of spinal and combined spinal epidural anesthesia for the operation were similar for both groups. Epidural dosing occurred in 6 patients in the active warming group and 4 in the passive warming group (*p* = 0.51, chi square). In addition, there was no difference in the time from entry to the operating room to neuraxial block and Foley catheter to skin incision and delivery. There was no difference in crystalloid administration or quantitative blood loss.

**Table 1.** Patient, Obstetric, Anesthetic And Surgical Variables.

	Forced Air Warming (n = 60)	Passive Warming (n = 60)	*P* value
Age (years)	29.3 ± 5.1	29.4 ± 6.4	0.94
Weight (kg)	96.4 ± 29.9	98.2 ± 26.8	0.73
BMI (kg/m^2^)	35.9 ± 10.1	36.5 ± 8.8	0.70
ASA Score	2 (2,2)	2 (2,2)	0.69^KS^
Gestational Age (days)	273 (266,274)	273 (271,275)	0.89^KS^
Operation (repeat/BTL/twins)	41/14/5	47/11/2	0.35^chi-square^
Spinal/CSE (*n*)	21/39	23/37	0.84^chi-square^
Total Operating Room Time (min)	110.0 ± 33.5	108.5 ± 31.3	0.77
In Room to Foley Temp (min)	24.6 ± 9.5	25.7 ± 9.9	0.54
In Room to Skin Incision (min)	37.5 ± 11.1	38.7 ± 11.6	0.52
Skin Incision to Delivery (min)	13 (9,17)	14 (14,19)	0.92^KS^
Uterine Incision to Delivery (min)	2 (1,2.75)	1 (1,2)	0.98^KS^
Crystalloid Volume (ml)	1832 ± 489	1745 ± 474	0.32
Quantitative Blood Loss (ml)	675 (500,875)	575 (370,785)	0.25^KS^

Data are expressed as average ± standard deviation or median (25%tile, 75%tile) or number (N/N/N).

Unless indicated a Student’s t test was used to analyze differences between groups.

*KS* Kolmogorov Smirnoff two sample test of non-parametric data.

*CSE* combined spinal epidural.

*BTL* bilateral tubal ligation.

Table 2 details the temperature differences. There was a small but significant difference in the preoperative oral temperature before presentation to the operating room in the active warming group. The time after operating room entry to obtain initial Foley catheter temperatures was similar (24.6 ± 0.4 active warming vs 25.7 ± 9.8 passive warming, min, *p* = 0.63) as were the initial temperatures (36.6 ± 0.4 active warming vs 36.5 ± 0.4 passive warming, **°**C, *p* = 0.38). There was no difference in the number of patients with an initial core temperature > 36 **°**C (59 and 56, active warming vs. passive warming respectively). There were subsequent differences with the lowest and final temperatures in the operating room (Table 2, *p* <  0.005). In addition to differences in the lowest temperature, there was a difference in distribution with the active warming group maintaining temperatures above 36 **°**C (*n* = 18 vs 5) and the number of patients < 35 **°**C in the passive warming group (*n* = 17 vs 4 active warming, *p* <  0.001 2X4 FE contingency table). At the end of surgery, the passive warming group continued to have 13 parturients < 35 **°**C and 5 > 36 **°**C while the active warming group had 0 < 35 **°**C and 19 > 36 **°**C (*p* <  0.001 2X4 FE contingency table). These measurements indicate that the loss of temperature in the operating room was minimized and the rewarming improved by active warming during the procedure. The time from the final Foley temperature in the operating room to the initial oral temperature in the recovery area was of short duration (6 ± 4.3 vs 7 ± 4.7 min, active warming vs passive warming). There were small statistically significant differences in the oral temperature measurements (36.6 ± 0.3 and 36.3 ± 0.3, *p* <  0.001 active warming vs passive warming) and no difference in the time to discharge from recovery. In Table 2, we also report the effect size of the temperature differences using the standardized mean difference (active warming mean – passive warming mean)/pooled SD) [29]. The temperature differences in the preoperative and lowest intraoperative temperature reveal a moderate effect size (0.07) while the temperature differences at the end of surgery indicate a large effect. These differences were also evident in the odds ratio of 5.1 for inadvertent perioperative hypothermia in the passive warming group. Using the large and moderate effect size the number needed to treat to obtain the benefit from active warming vs passive warming is 2 to 5 [29].

**Table 2.** Temperature Variables.

	Forced Air Warming (*n* = 60)	Passive Warming (*n* = 60)	Effect Size Estimate	*P* value
Preoperative Temperature (**°**C)	36.8 ± 0.3	36.6 ± 0.3	0.7*	< 0.005
Operating Room Temperature (**°**C)	18.9 ± 1.0	18.7 ± 1.2	0.2*	0.19
Initial Foley Core Temperature (**°**C)	36.6 ± 0.4	36.5 ± 0.4	0.3*	0.38
Lowest Foley Core Temperature (**°**C)	35.6 ± 0.5	35.3 ± 0.5	0.7*	< 0.005
Final Foley Core Temperature (**°**C)	36.0 ± 0.5	35.4 ± 0.5	1.2*	< 0.005
Intraoperative Hypothermia (< 36 **°**C, n, %)	41, 68%	55, 92%	5.1^+^	< 0.005
Recovery Room Temperature (**°**C)	36.6 ± 0.3	36.3 ± 0.3	0.7*	< 0.005

Data are expressed as average ± standard deviation, number (N), or percentage.

* = Standardized Mean Difference

+ = Odds Ratio for temperature <  36 °C without forced air warming

Table 3 details the neonatal outcomes with no differences in birth weights, Apgar scores, or umbilical artery pH or base excess. With only a few exceptions, the umbilical artery measurements were within normal limits (pH < 7.1, none in the active warming and, 3 in passive warming group, chi square p = 0.24) [16, 30]. Apgar scores < 7 at 1 or 5 min were equivalent in both groups (active warming vs. passive warming n = 3 vs. 6 at 1 min *p* = 0.32 and 2 vs. 0 at 5 min *p* = 0.49).

**Table 3:** Neonatal Outcomes

	Forced Air Warming (*n* = 60)	Passive Warming (*n* = 60)	*P* value
Birth Weight (kg)	3.2 ± 0.6	3.3 ± 0.5	0.13
Apgar 1 min	9 (8,9)	9 (8,9)	0.99 ^KS^
Apgar < 7 at 1 min (n)	3	7	p = 0.32 ^chi-square^
Apgar 5 min	9 (9,9)	9 (9,9)	1.0 ^KS^
Apgar < 7 at 5 min (n)	2	0	p = 0.49 ^chi-square^
Umbilical Arterial Blood pH	7.24 ± 0.06	7.23 ± 0.07	0.18
Umbilical Arterial Blood pH < 7.1 (n)	0	3	p = 0.24 ^chi-square^
Base Excess	−3.0 ± 2.3	− 3.8 ± 3.1	0.16

Data are expressed as average ± standard deviation or median (25%tile, 75%tile).

Unless indicated an unpaired two tailed Student’s t test was used to analyze differences between groups.

KS Kolmogorov Smirnoff two sample test of non-parametric data.

In addition to neonatal outcomes, we reviewed all subsequent health care notes. All patients had outpatient post discharge post-partum visits (3–4 weeks post-partum) that documented wound status. There were no patients with either superficial or deep wound infections. In the passive warming group there was one patient with a wound seroma (not described) and one with slight induration that resolved without therapy. In contrast, in the active warming group there were 5 patients with small (< 1 cm) area of wound dehiscence and 2 patients with wound seromas and dehiscence measuring 1 and 4.5 cm. No patient received antibiotic therapy or had any apparent extra clinic or emergency department visits.

The datasets used and/or analyzed during the current study are available from the corresponding author on reasonable request.

## Discussion

There are three findings related to active warming in addition to warmed IV/irrigation fluids before and during elective repeat cesarean delivery in this project. First, prewarming had a small positive impact on preoperative temperature that did not translate into higher initial core temperatures in the operating room. Second, active warming was associated with higher intraoperative and immediate postoperative temperature measurements. Third, the incidence of inadvertent perioperative hypothermia was less in the active warming group.

The use of spinal anesthesia with hyperbaric bupivacaine and morphine provides several challenges during cesarean delivery. The deleterious effects of spinal anesthesia on cutaneous vasodilation, redistribution of core body heat, and overall heat loss have been previously described [7, 8]. The use of intrathecal morphine further exacerbates this effect [31]. In contrast, a continuous phenylephrine infusion, [25] the administration of fluids using fluid warmers [32–35] or a warming cabinet (37–45 **°**C) [10, 14, 36, 37] have a positive effect on maternal temperature during cesarean delivery. While previous studies have recorded positive effects with these methods, those efforts have been insufficient in preventing inadvertent perioperative hypothermia [5, 10, 11, 13–16, 33–36].

In this retrospective observational cohort study, − in addition to standard intravenous/irrigation fluid warming – we evaluated preoperative and intraoperative active warming to prevent/limit thermal redistribution and heat loss. We used the Bair Paws™ warming gown for preoperative warming because it is a standard part of perioperative temperature regulation in our institution and allows mothers to choose their comfort level representing how patients use the device in real time. We used the Bair Hugger™ full access underbody blanket during the procedure because (1) lower extremity active warming studies have not been effective, [5, 38] (2) upper body gowns billow significantly during use, encroaching on the mother’s face and limiting access to the arms during the procedure, and (3) the underbody blanket provides for improved access for infant skin-to-skin bonding immediately after delivery while continuing to deliver warmth to mother and child.

Our results are similar to other previous studies evaluated the efficacy of active warming before neuraxial block administration in conjunction with upper body active warming during the procedure [9, 18, 28]. de Bernardis studied 40 patients undergoing elective cesarean delivery and evaluated the Bair Paws™ gown in both the preoperative (total body) and intraoperative arena (upper body) [18]. The spinal anesthesia and the volume of warmed fluids (37 **°**C) was similar to our population but the duration of surgery was shorter at 60 min and BMI range smaller (29–30). Baseline temperatures (digital tympanic) were similar, and at the end of surgery temperatures were slightly higher than in our investigation (36.2 **°**C vs. 36.0 **°**C) but the magnitude of changes between groups were similar. Horn found that 15 min of upper extremity active warming with fluid warming before an epidural anesthetic was effective in maintaining a normal temperature (tympanic thermocouple probe) in patients undergoing elective cesarean delivery [9]. The study, however, employed only epidural anesthesia and is limited by the practicality of performing preanesthetic active warming in the operative suite. Finally, in an evaluation of isolated preoperative active warming for 20 min using a full body system in addition to warmed intravenous fluids did not alter hypothermia or maternal comfort [28]. Based on the slight increase in temperature after use of the Bair Paws™ before surgery and no difference in the initial Foley temperatures that aspect of active warming may provide minimal if any benefit.

There were no differences in the secondary neonatal indicators of poor outcome (Apgar scores, placenta arterial pH, base deficit) [30]. This finding is consistent with the work of Cobb [5] and Chung [14] who reported no differences in umbilical vein pH or Apgar scores and Grant [16] who also reported no difference in umbilical artery pH or Apgar scores in their trials despite improved thermal management. This is in slight contrast to the study of active warming by Horn [9]. While they observed differences in the core temperature between groups, the Apgar scores were similar despite significant aberrations in placental vein pH and base excess with passive warming.

As with any intervention or change in practice the benefits must be balanced with the time/effort to implement a change and costs. The time/effort of the logistics chain, using a preoperative warming gown and placing an underbody warming blanket on the operating room table are negligible. However, there are capital investment costs ($1750.00 each - 3 M suggested list price) and the variable costs of the warming gown and underbody blanket for each procedure ($14.00 and $16.00 respectively - 3 M suggested list price). However, actual costs are dependent on negotiated contracts which can reduce acquisition costs by as much a 50%.

A criticism of this report is the lack of randomization and blinding. However, quasi-experimental designs are useful when internal validity threats (history, maturation, observation, and instrumentation) are addressed [20]. Though these challenges are more prominent in long term studies, they also need to be considered here. Historical and maturation challenges were minimal as this evaluation was conducted over a relatively short time frame (9 months) with a core faculty group providing both anesthetic and obstetrical services. This resulted in similar patient characteristics, anesthetic and surgical procedural times, equivalent environmental management, fluid administration and blood loss. In addition, there were no changes in measurement techniques over the time. Despite the interrupted time series design, it is unlikely that within the time frame of analysis there was a systematic variable or natural trend for patients to have decreased loss of temperature and in increased warming other than the associated addition of active warming to our passive warming methods.

Because this was a pragmatic application of change we did not control or systematically measure how patients used the Bair Paws™ warming gown before surgery. In daily practice patients regulate temperature delivery based on thermal comfort and not maximizing heat transfer which at times is intolerable [28].

Another criticism would be maintaining the intraoperative temperature at an average of 18.5 **°**C. While increased intraoperative temperature at 20–23 **°**C may or may not further improve thermal management it is known to increase surgeon discomfort [17, 39]. When approaching this project we decided to evaluate the addition of active warming as the highest probability for a beneficial impact prior to systematically altering the operating room temperature.

## Conclusion

In summary, we report a pragmatic initiative to reduce inadvertent perioperative hypothermia during cesarean delivery. As outlined, warmed intravenous and irrigation fluids, combined with pre and intraoperative active warming during elective repeat cesarean delivery maintained maternal temperature better with a decreased incidence of inadvertent perioperative hypothermia (< 36 °C). The primary outcomes of temperature and inadvertent perioperative hypothermia at the end of surgery are driven by total body heat and the spinal anesthetic which causes vasodilation with redistribution and reduction in the core temperature. The longer surgical times in our population may have impacted the positive rewarming in the active warming group and may not be applicable in facilities with shorter surgical times. We did not observe benefits in the secondary neonatal and maternal outcomes. However, this evaluation is underpowered to detect differences in those outcomes.

## Data Availability

The datasets used and/or analyzed during the current study are available from the corresponding author on reasonable request.

## Abbreviations

C: Centigrade
SD: Standard deviation
FE: Fisher Exact
BMI: Body mass index
ASA: American Society of Anestheisologists
KS: Kolmogorov Smirnoff two sample test of non-parametric data.
CSE: Combined spinal epidural
BTL: Bilateral tubal ligation
